# Are profiles of job insecurity associated with health‐related indicators among faculty in Swedish academia?

**DOI:** 10.1111/sjop.13064

**Published:** 2024-08-26

**Authors:** Anna S. Tanimoto, Anne Richter, Aleksandra Bujacz, Petra Lindfors

**Affiliations:** ^1^ Department of Psychology Stockholm University Stockholm Sweden; ^2^ Department of Learning, Informatics, Management and Ethics Karolinska Institutet Solna Sweden

**Keywords:** Psychosocial work environment, subjective job insecurity, higher education institutions, health and well‐being, person‐oriented methods

## Abstract

Job insecurity is a work stressor associated with various health‐related impairments. As concerns about the ubiquity of job insecurity in academia have become increasingly prominent, the potential implications of job insecurity for the health and well‐being of faculty require attention. Specifically, these implications may vary between groups within academia, yet little is known about such variations, particularly with respect to different indicators of health and well‐being. This study aims to identify and examine profiles of job insecurity (including quantitative and qualitative dimensions) in relation to exhaustion, depressive symptoms, well‐being, and work–family conflict among faculty in Sweden. Self‐reports in questionnaires were collected in 2021 from a representative sample of faculty, with a doctoral degree, working in Swedish public higher education institutions (*N* = 2,729 respondents; 48% women; average age: 50 years; 82% born in Sweden). Latent profile analysis was conducted to identify profiles of job insecurity, followed by statistical comparisons on demographic covariates and health‐related indicators across profiles. The latent profile analysis revealed five job insecurity profiles: the moderately insecure (n = 215), the secure (n = 1777), the secure; quality‐concerned (n = 406), the insecure; employment‐concerned (n = 177), and the insecure (n = 154). Twelve percent of the sample was identified as vulnerable, particularly the insecure profile, where these individuals may be most at a risk for exhaustion disorder and depression. Among faculty in Sweden, quantitative and qualitative dimensions of job insecurity appear to be closely connected, with the qualitative dimension seemingly more informative for health‐related indicators.

## INTRODUCTION

Increasing concerns about job insecurity (JI) in academia in Western countries have prompted investigations indicating potential consequences for faculty health (Kinnunen, Mäkikangas, Mauno, De Cuyper & De Witte, 2014; Nicholls, Nicholls, Tekin, Lamb & Billings, 2022; Urbanaviciute, Roll, Tomas & De Witte, 2021). While those in academia are tasked with the responsibility to educate and innovate, little is known about fixed‐term contracts and JI perceptions among faculty. Specifically, the concurrent concerns of job loss and deteriorating conditions at work, and their combined health‐related implications, remain unclear. Moreover, context is crucial given that employment protection legislation, and welfare systems can influence employment contracts and JI (Erlinghagen, 2008; Lee, Huang & Ashford, 2018; Sverke, Låstad, Hellgren, Richter & Näswall, 2019). For instance, in Sweden there is more fixed‐term employment in academia (i.e., 27%) than in any other sector (Swedish Higher Education Authority, 2023), indicating that JI in academia is a concern. Considering this, the present study aimed to: (1) investigate whether it is possible to identify unique profiles of JI, using theoretically‐defined threats of job loss and threats of deteriorating job conditions; (2) investigate whether such profiles are associated with different positive and negative health‐related indicators; and (3) provide insight into the extent of JI experiences among faculty across Swedish academia.

## WHAT IS JOB INSECURITY?

JI can be operationalized in two ways: objective and subjective JI. Typically, objective JI refers to the type of employment contract an individual has (e.g., permanent or fixed‐term). Employees with fixed‐term contracts are considered objectively insecure (Pearce, 1998) given the lack of continuity in the job. Most of the literature on JI in academia covers objective JI. Indeed, across the academic sector, fixed‐term contracts have been on the rise (OECD, 2021) and a recent meta‐synthesis of qualitative studies identified faculty concerns regarding uncertainty surrounding temporary contracts as an important theme (Nicholls *et al*., 2022).

Subjective JI reflects individual perceptions of JI and is not contingent upon the type of contract (Greenhalgh & Rosenblatt, 1984). Certainly, one's contract type may influence one's subjective JI, but any employee may experience JI. Thus, the operationalization of subjective JI focuses on individuals' experiences. Distinguishing between objective and subjective JI is important as studies show that subjective JI is associated with more health‐related consequences (Van den Tooren & De Jong, 2014). This provides a rationale for focusing on subjective JI.

JI refers to “a perceived threat to the continuity and stability of employment as it is currently experienced” (Shoss, 2017, p. 1914). Most extant JI research makes a theoretical distinction between the perceived threat of job loss, and the perceived threat of losing valued qualities in the current job (Hellgren, Sverke & Isaksson, 1999). These threats are distinguished as quantitative and qualitative JI, respectively. Quantitative JI reflects concerns or worries about losing one's current job so the continuity of the job itself is of central concern. Qualitative JI, however, does not reflect worries about the continuity of the job; it reflects concerns about the continuity of certain features, qualities, and conditions of the current job. Thus, qualitative JI reflects concerns that the conditions of the current job will deteriorate in the future.

## WHY IS IT DETRIMENTAL?

Assuming that job‐related losses are undesirable, JI implies a perceived threat. Theoretically, this can be referred to as anticipatory stress (Frankenhaeuser, 1981). Consequently, the possibility of job‐related loss, (an unwanted outcome) may trigger individual stress reactions due to the uncertainty of the outcome. Not knowing when or if an unwanted event will occur may be stressful given that individuals facing such situations are unable to take appropriate action while the outcome remains unknown. Conversely, when individuals learn of an unwanted outcome, the result is known, allowing for the initiation of stress‐reducing behaviors. While a high education level may connote health advantages (Marmot, 2005; Olsen, Lindberg & Lamu, 2020), experiences of JI may contribute to a social disadvantage for faculty, with potentially negative implications for their health. The uncertainty associated with perceived JI makes it a work stressor which contributes to stress reactions, and if prolonged this may increase allostatic load and impair health (McEwen, 1998), even among the highly educated. Importantly, health‐related implications may involve both impaired mental health and well‐being (Låstad, Tanimoto & Lindfors, 2021).

Besides poor health, JI may engender other consequences, including work–family conflict (WFC). Work stress may cause irritability or preoccupation with work, potentially impairing individuals' capacity to fulfill responsibilities within the family domain (Frone, Russell & Cooper, 1992; Greenhaus & Beutell, 1985). Thus, work stressors may contribute to an interference between work and family domains if job loss puts economic strain on individuals' households or if more time is spent at work in attempts to counteract potential job loss (Voydanoff, 2005). Theoretically, JI can be assumed to have implications extending beyond the work domain.

## EMPIRICAL FINDINGS

Findings consistently support the relationship between JI and various adverse health‐related consequences. Meta‐analyses emphasize the negative effects of JI on indicators of physical and mental health (Cheng & Chan, 2008; Jiang & Lavaysse, 2018; Llosa, Menéndez‐Espina, Agulló‐Tomás & Rodríguez‐Suárez, 2018; Sverke, Hellgren & Näswall, 2002) and longitudinal findings link JI to impaired health and well‐being (Burgard, Brand & House, 2009; De Witte, Pienaar & De Cuyper, 2016). JI has been related to burnout (Blom, Richter, Hallsten & Svedberg, 2015; Boone, Vander Elst, Vandenbroeck & Godderis, 2022; Kinnunen *et al*., 2014), depressive symptoms (Blom *et al*., 2015; Llosa *et al*., 2018), and reduced psychological well‐being (Låstad *et al*., 2021). Also, findings support the relationship between JI and spillover from work to family domains, with JI being associated with WFC (Jiang & Lavaysse, 2018; Mauno, Cheng & Lim, 2017; Voydanoff, 2005).

Existing research tends to focus on the effects of quantitative JI (Lee *et al*., 2018). This may relate to difficulties distinguishing between quantitative and qualitative JI or that quantitative JI is viewed as more detrimental. Research covering contextual factors including unemployment rates or social welfare systems seem to prioritize quantitative JI (Lee *et al*., 2018). Still, there is an increasing interest in qualitative JI as the concerns of changing and deteriorating working conditions have become more salient, thus bringing forth questions as to the various consequences which may arise. Importantly, studies examining the effects of both dimensions (in separate statistical models), suggest that quantitative and qualitative JI may be equally harmful (De Witte, De Cuyper, Handaja, Sverke, Näswall & Hellgren, 2010; Richter, Näswall, Bernhard‐Oettel & Sverke, 2014). When the two dimensions have been studied simultaneously (in the same statistical model; e.g., Chirumbolo, Urbini, Callea, Lo Presti & Talamo, 2017; Nawrocka, De Witte, Brondino & Pasini, 2021), findings suggest that qualitative JI may be more important for health and well‐being (Chirumbolo *et al*., 2017; Nawrocka *et al*., 2021). This underscores the importance of including qualitative JI when investigating health‐related indicators.

Importantly, there is an ongoing theoretical discussion about the interrelations between the two JI dimensions (De Witte, Vander Elst & De Cuyper, 2015). Some argue that quantitative JI should imply greater loss than qualitative JI, as this would involve job loss and all ensuing characteristics (De Witte *et al*., 2015). Thus, quantitative JI should encompass qualitative JI when investigating the two dimensions simultaneously. While this notion is supported cross‐sectionally (Chirumbolo *et al*., 2017), prospectively, qualitative JI appears to precede quantitative JI (Nawrocka *et al*., 2021). While temporal differences between study designs may explain these inconsistencies, mixed findings may also result from population differences. Among certain groups, the dimensions may be strongly associated, while in other populations, associations may be weaker. As such, the relationship between the dimensions may be population specific. This warrants a contextualized focus on specific populations. Such a focus can be argued to benefit from a person‐oriented approach, which accounts for JI perceptions more holistically, simultaneously providing a more population‐specific understanding of the interplay between the quantitative and qualitative dimensions.

## A PERSON‐ORIENTED APPROACH

Person‐oriented approaches provide a complementary perspective to the traditional and dominating variable‐oriented approaches. While in variable‐oriented approaches, the units of interest are the phenomena under investigation (i.e., the variables), the person‐oriented approach focuses on individuals (or cluster groups of individuals), treating them as individual entities (Bergman & Lundh, 2015; Bergman & Magnusson, 1997) Moreover, person‐oriented approaches are considered typological methods that make use of the variability between individuals within a larger, heterogenous sample, to identify patterns characterizing subpopulations or homogenous groups of individuals. These patterns are based on certain shared or similar characteristics or experiences to establish theoretically and practically meaningful but still distinctive types or profiles (Bergman & Trost, 2006). These profiles may then be examined in relation to other factors of interest, including demographics. While profiles do not account for unique individual experiences, person‐oriented approaches come closer to understanding how various individuals experience a specific psychological phenomenon, whereas the variable‐oriented approach overlooks individuals to focus on aggregate explanations between variables. Importantly, in allowing for a flexible modeling of complex patterns, the person‐oriented approach extends the prevailing analysis of interactions between variables, which may expand existing theory.

JI research has a long tradition of variable‐oriented methods, yielding conclusions based on treating study samples as homogenous. Yet in 2014, a Finnish study elucidated the strengths of a person‐oriented approach by identifying eight unique trajectories of JI among university staff (Kinnunen *et al*., 2014). Over a two‐year period, faculty belonging to a stable, moderately high JI type, repeatedly reported high exhaustion (Kinnunen *et al*., 2014). However, the identified trajectories were limited to quantitative JI. Recent person‐oriented research, including both quantitative and qualitative dimensions, suggests that some individuals report patterns of quantitative and qualitative JI that are not associated with adverse health. Yet, others report patterns of quantitative and qualitative JI where such patterns do have negative implications for employee health and well‐being (Ghezzi, Ciampa, Probst *et al*., 2022; Låstad *et al*., 2021; Urbanaviciute, Lazauskaite‐Zabielske & De Witte, 2021). Such person‐oriented findings are novel in the vast JI research, and necessary to pinpoint vulnerable individuals who may benefit from interventions to mitigate JI, in academia and elsewhere.

## AIMS AND CONTRIBUTIONS

Based on existing theory, this study aims to identify unique profiles of JI, including its quantitative and qualitative dimensions, through latent profile analysis, and to investigate associations with health‐related indicators among faculty in Sweden. This study seeks to: (1) clarify variations in the concurrent interplay of quantitative and qualitative JI; (2) explore differences in health‐related indicators including exhaustion, depressive symptoms, well‐being, and WFC across profiles; and (3) examine the extent of JI perceptions in Swedish academia. In doing so, this study makes several contributions. With a person‐oriented approach to JI, we empirically contribute to a field that traditionally takes a variable‐oriented approach. The person‐oriented approach allows for a closer investigation of if and how the two dimensions occur simultaneously among similar individuals. Specifically, this adds to the theoretical discussion regarding the interrelations between quantitative and qualitative dimensions, and provides contributions regarding which dimension may be more important for health. Until now, only a few studies have taken this approach.

Another noteworthy contribution regards the context and sample. As contextual factors may affect levels of both quantitative and qualitative JI (Erlinghagen, 2008; Lübke & Erlinghagen, 2014; Sverke *et al*., 2019), such factors should be considered. Importantly, knowledge of the concurrent interplay of quantitative and qualitative JI, particularly in highly educated groups, including faculty in Swedish academia, remains limited. With a representative sample of faculty working at higher education institutions in Sweden, this study contributes to sector‐specific investigations of JI, adding to a general understanding of the extent of subjective JI among those working in Swedish academia.

## MATERIALS AND METHODS

### Data collection, procedure, and participants

Self‐reports in questionnaires were collected by Statistics Sweden in the fall of 2021. A representative sample of 7446 women and men, between the ages of 28 and 65, with a doctoral degree, working in Swedish higher education institutions were invited to participate. An invitation with a code to the online survey was mailed, followed by two reminders (after 2 and 4 weeks). In addition to information regarding the overall study, protection of personal information, and data handling, participants were asked to provide their informed consent. The Swedish Ethical Review Authority approved this study (2020/07195). The overall response rate was 39%.

This study was based on a select set of data from a comprehensive questionnaire directed towards faculty in Swedish academia. The study only included individuals reporting that their HEI employment was their main activity (i.e., individuals combining part‐time work with an income from pension or parental benefits were excluded). This resulted in an effective sample of *n* = 2,729, including 48% women, aged between 30 and 66 years old (mean age 50), 82 percent of whom were born in Sweden.

### Measures

Perceived JI was measured with three quantitative JI items (Låstad, Berntson, Näswall, Lindfors & Sverke, 2015), and four qualitative JI items (Fischmann, De Witte, Sulea, Elst, De Cuyper & Iliescu, 2022; Låstad *et al*., 2015). Quantitative items targeted perceived risk of job loss, example item: “I'm afraid I will lose my job.” Qualitative items covered concerns about losing valued features of the job, example item: “I think that my job will change for the worse in the near future.” Response alternatives ranged from 1 – *strongly disagree* to 5 – *strongly agree*.

A single item measured exhaustion (Eriksen, Ihlebæk & Ursin, 1999; Hultell & Gustavsson, 2010): “During the past month, have you felt exhausted?” with response alternatives ranging from 1 – *not at all* to 5 – *extremely*.

A six‐item version of the symptom checklist‐core depression (SCL‐CD6) scale was used to measure depressive symptoms (Magnusson Hanson, Westerlund, Leineweber, *et al*., 2014; example item: “During the past week, how much have you been feeling that everything is an effort?”). Response alternatives ranged from 1 – *not at all* to 5 – *extremely*.

The seven‐item version of the Warwick‐Edinburgh mental well‐being scale (WEMWBS; Stewart‐Brown, Tennant, Tennant, Platt, Parkinson & Weich, 2009) measuring hedonic and eudaimonic well‐being over the past two weeks was used (example item: “I have been feeling optimistic about the future.”). Response alternatives ranged from 1 – *all of the time* to 5 – *none of the time*. Responses were recoded with high scores reflecting better well‐being.

Work‐life conflict was measured with a single‐item based on Frone *et al*. (1992): “How often does your work influence your personal life in a negative way?” Response alternatives ranged from 1 – *almost never* to 5 ‐ *almost always*.

Demographics included sex (man = 0; woman = 1), age, years since receiving a Ph.D., civil status (0 = single; 1 = partnered), children living at home under the age of 18 (0 = none; 1 = yes), and contract type (0 = fixed‐term; 1 = permanent) as JI perceptions may be influenced by demographic characteristics and type of employment contract (Lee *et al*., 2018). Similarly, these demographics relate to various indicators of health and well‐being (Lindfors, Berntsson & Lundberg, 2006; Magnusson Hanson *et al*., 2014).

### Data preparation and statistical procedures

#### Normality checks and missing data

To check the normal distribution of the data, skewness and kurtosis on the raw scores were examined, and residuals were plotted, providing visual confirmation of normality. Raw scores were below three for skewness and below ten for kurtosis, indicating no severe skewness nor kurtosis on the study variables (Kline, 2016). Additionally, no correlation between study variables exceeded 0.85, indicating that multicollinearity was not detected (Weston & Gore, 2006). Missing data analyses revealed that the extent of “missingness” on study variables was less than 1%. Missingness was handled with full information maximum likelihood estimation for the analyses conducted in Mplus (Version 8.7).

#### Measurement model

Two measurement models were tested: a seven‐factor model and a six‐factor model. The seven‐factor model included a general JI factor, one specific factor for quantitative JI, one specific factor for qualitative JI, and latent factors for exhaustion, depression, well‐being, and WFC. The six‐factor model consisted of one quantitative, and one qualitative JI factor in addition to the same four health‐related factors. These two models were compared using goodness of fit statistics with the following cut‐offs: CFI > 0.90, TLI > 0.90, SRMR < 0.08, and RMSEA < 0.08 (Hu & Bentler, 1999). Additionally, Akaike information criterion (AIC) and Bayesian information criteria (BIC), where lower values indicate better fit (Kline, 2016), provided additional information for model comparison. Following the selection of the model, factor scores^1^ were saved for use in the profile enumeration procedure in order to avoid issues of nonconvergence (Morin, McLarnon & Litalien, 2020). Factor scores for the health‐related indicators were also saved for the auxiliary variable analyses.

#### Profile enumeration

Six models were estimated, including two to seven profiles. A step‐wise procedure (Asparouhov & Muthén, 2012) tests each profile solution for its fit and compares it to the previous profile (*k*−1). The first step involves obtaining and replicating the best loglikelihood for *k* classes. The second step compares the *k*−1 loglikelihood to *k* classes loglikelihood and if the loglikelihood difference between the two is significant, this suggests that the *k*−1 class model can be rejected for the *k* class model. The third step obtains the bootstrapped likelihood ratio test (BLRT) to compare the *k*−1 class model to the *k* class model. A significant *p*‐value indicates that the *k* class model improves model fit. Criteria used to assess the optimal profile solution include: AIC, BIC, sample‐size adjusted BIC (SABIC), the Lo–Mendell Rubin adjusted likelihood ratio test (LMR), the BLRT, and the percent of the sample belonging to each profile (where a minimum of 5% is recommended; Stanley, Kellermanns & Zellweger, 2017). While entropy should not be used for solution selection, scores approaching 1 indicate higher accuracy regarding overall classification (Morin *et al*., 2020).

#### Auxiliary variable analyses

Profile comparisons were conducted for demographics and health‐related indicators. For categorical covariates, chi‐square tests and pair‐wise comparisons were performed through the DCAT method in Mplus (Lanza, Tan & Bray, 2013), while continuous covariates and health‐related indicators were compared using the equality of means test through BCH automatic estimation in Mplus (Asparouhov & Muthén, 2021).

## RESULTS

### Descriptive statistics

Table 1 shows variable means, Pearson correlations, and Cronbach's alphas for complete cases (*n* = 2,685).

**Table 1:** Means, standard deviations, Pearson correlations, and Cronbach's alphas (on the diagonal) for study variables (complete cases)

Variable	Mean (SD) or %	1.	2.	3.	4.	5.	6.	7.	8.	9.	10.	11.	12.
1. Sex (woman)	48%	–											
2. Age	50 (8.84)	−0.03	–										
3. Ph.D. Tenure	14 (8.19)	−0.17***	0.70***	–									
4. Civil status (partnered)	86%	−0.06**	−0.01	0.02	–								
5. Children (yes)	51%	−0.01	−0.44***	−0.27***	0.21***	–							
6. Contract type (permanent)	87%	−0.02	0.40***	0.39***	0.01	−0.06**	–						
7. Quant JI	1.69 (1.12)	0.02	−0.31***	−0.26***	−0.01	0.09***	−0.44***	0.96					
8. Qual JI	2.37 (1.15)	−0.00	−0.23***	−0.20***	−0.02	0.08***	−0.20***	0.53***	0.92				
9. Exhaustion	2.43 (1.23)	0.07***	−0.20***	−0.19***	−0.03	0.09***	−0.13***	0.26***	0.39***	–			
10. Depressive s.	13.99 (5.56)	0.08***	−0.20***	−0.20***	−0.06**	0.08***	−0.14***	0.31***	0.50***	0.75***	0.90		
11. Well‐being	3.83 (0.51)	−0.01	0.18***	0.16***	0.09***	−0.04	0.13***	−0.32***	−0.46***	−0.49***	−0.66***	0.82	
12. WFC	2.97 (1.03)	0.11***	−0.07***	−0.08***	0.00	0.05*	−0.06**	0.23***	0.40***	0.49***	0.53***	−0.41***	–

*Notes*: *n* = 2685 (complete cases). Quant JI = Quantitative Job Insecurity; Qual JI = Qualitative Job Insecurity; Depressive s. = Depressive Symptoms; WFC = Work–Family Conflict.

*** *p* < 0.001,

** *p* < 0.01,

* *p* < 0.05, two‐tailed.

### Measurement model

Two models were tested: the seven‐factor model (including a general factor, a quantitative factor, and a qualitative factor) and the six‐factor model. Standardized factor loadings for the seven‐factor model ranged from 0.35 to 0.91 (*p* < 0.001), while loadings for the six‐factor model ranged from 0.53 to 0.97 (*p* < 0.001). Quantitative and qualitative JI factors were moderately correlated, *r* = 0.57 in the six‐factor model. Table 2 displays fit statistics for the competing models. Based on the model statistics and information criteria, the six‐factor model was selected.

**Table 2:** Comparison of two measurement models

Model	df	*χ* ^2^	CFI	TLI	SRMR	RMSEA	AIC	BIC
7‐Factor	188	2110.87***	0.94	0.93	0.06	0.06	132067.85	132582.17
6‐factor	196	2014.79***	0.94	0.93	0.04	0.06	131966.09	132433.12

*Notes:* df = Degrees of Freedom.

*** *p* < 0.001.

### Selection of profile solution

**Fig. 1:**
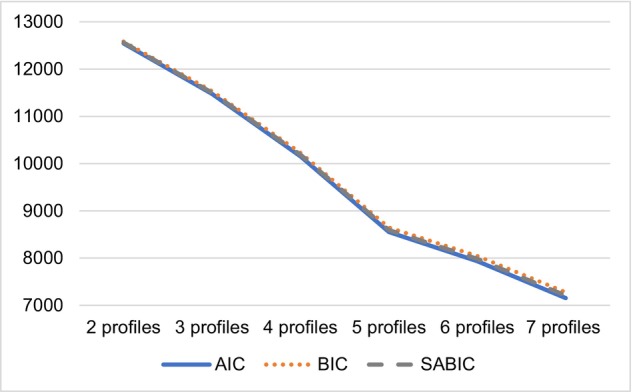
Information criteria elbow plot for two to seven profile solutions.

Table 3 contains model fit statistics for the profile solutions. The information criteria (AIC, BIC, SABIC) steadily decreased with each new profile solution, and significant *p*‐values for LRT and BLRT indicated that the number of classes had not yet been reached. This can occur when sample sizes are large (Marsh, Lüdtke, Trautwein & Morin, 2009). In such cases an elbow plot can be informative for the selection of the profile solution (Fig. 1). Examining the profile solutions where the smallest profile percentage was greater than or equal to 5% showed that the four and five‐profile solutions seemed preferable. Still, the five‐profile solution yielded lower values on information criteria, suggesting that of the two, this solution better approximated the data. Furthermore, the five‐profile solution yielded a sufficient number of profiles to reveal profiles with balanced and unbalanced levels of the profile indicators.

**Table 3:** Enumeration statistics for two to seven latent profile models

Model (*k*)	AIC	BIC	SABIC	#fp	Entropy	LRT (*P*)	BLRT (*P*)	Latent profile proportions %
2‐profile	12544.09	12585.48	12563.23	7	0.96	<0.001	<0.001	81/19
3‐profile	11475.26	11534.38	11502.61	10	0.94	<0.001	<0.001	69/20/11
4‐profile	10152.53	10229.38	10188.08	13	0.98	<0.001	<0.001	15/6/66/13
**5‐profile**	**8551.79**	**8646.37**	**8595.54**	**16**	**0.99**	**<0.001**	**<0.001**	**8/65/15/6/6**
6‐profile	7935.28	8047.60	7987.23	19	1.00	<0.001	<0.001	63/4/8/6/6/13
7‐profile	7153.40	7283.46	7213.55	22	1.00	<0.001	<0.001	4/6/3/63/7/6/11

*Notes:* Bold: selected profile solution. *N* = 2729. AIC = Akaike Information Criterion; BIC = Bayesian Information Criteria; SABIC = Sample‐Size Adjusted Bayesian Information Criterion; #fp = Number of Free Parameters; LRT = Lo–Mendell–Rubin Adjusted Likelihood Ratio Test; BLRT = BLRT = Bootstrapped Likelihood Ratio Test.

**Fig. 2:**
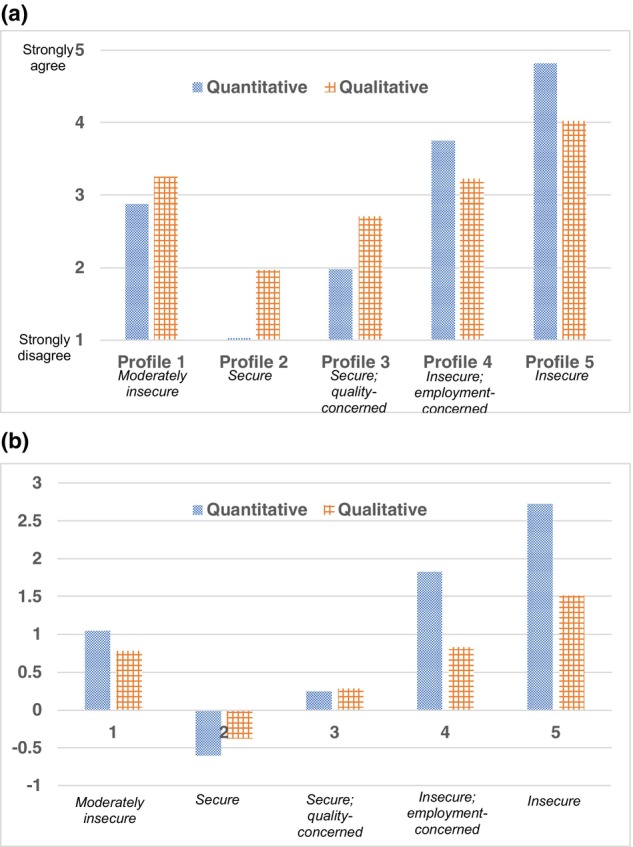
(a) Quantitative and qualitative job insecurity scale means by profile. (b) Quantitative and qualitative job insecurity factor means by profile.

Providing visual confirmation, Fig. 1 shows a steady decrease in information criteria tapering off after the five‐profile solution, which is why the five‐profile solution was chosen. For interpretability, the bar graph in Figure 2a displays the quantitative and qualitative *scale* means by profile. Figure 2b displays the factor means for quantitative and qualitative JI for the five‐profile solution. The estimates were as follows (factor means and standard deviations for quantitative and qualitative JI): Profile 1: 1.04 (0.16), 0.78 (0.71); Profile 2: −0.60 (0.08), −0.37 (0.81); Profile 3: 0.23 (0.18), 0.29 (0.73); Profile 4: 1.82 (0.21), 0.82 (0.71); Profile 5: 2.72 (0.16), 1.52 (0.61). As factor scores were used as the indicators in the profile enumeration procedure, these means reflect the average weighted score on each JI dimension, by profile.

### Profile descriptions

Profile 1, identified as the moderately insecure profile, accounted for 8% of the sample. It was characterized by moderate levels of quantitative and qualitative JI, where concerns regarding job features were slightly elevated as compared to concerns about the job itself (Fig. 2a, b). This profile involved moderately higher levels of quantitative and qualitative JI than average.

Profile 2, the secure profile, was the largest and accounted for 65% of the sample. This profile was characterized by an absence of quantitative JI and low qualitative JI scores. Those in this profile did not report any concerns of job loss per se, yet there was some concern regarding the possible loss of qualities. This profile had noticeably lower quantitative and qualitative JI than the other profiles.

Profile 3, referred to as the secure; quality‐concerned profile accounted for 15% of the sample. It was characterized by relatively low quantitative JI, but moderate qualitative JI. Concerns about the loss of the job itself were minimal, but concerns about negative changes were moderate. While this profile had higher JI than the secure profile, it had noticeably lower quantitative and qualitative JI than the remaining profiles.

Profile 4, named the Insecure; employment‐concerned profile, accounted for 6% of the sample and was characterized by relatively high quantitative, and moderate qualitative JI. Those in this profile reported concerns about job loss, and to a lesser extent, concerns about the job conditions. They reported higher quantitative JI than all other profiles except for Profile 5, and higher qualitative JI than Profiles 2 and 3.

Profile 5, the insecure profile, accounted for 6% of the sample and reflected both high quantitative and high qualitative JI. This profile was characterized by a high threat of job loss and relatively high concerns about losing valued job qualities. Those in this profile reported higher JI than all other profiles.

### Demographic comparisons by profile

Significant differences were found between profiles regarding continuous and categorical covariates (Table 4). Significant differences by age were found between most profiles: Profile 2 was older than all other profiles, Profile 1 was older than Profile 4, and Profile 3 was older than Profiles 4 and 5. Differences were found for all profiles regarding Ph.D. tenure except between Profiles 4 and 5. The overall test for sex was nonsignificant, but pairwise comparisons showed that Profile 4 included more men, while Profile 5 included more women. Similarly, the overall test for civil status was nonsignificant, but pairwise comparisons showed more single individuals in Profile 4 than in Profiles 1, 2, and 5. As for children, individuals in Profile 2 had significantly fewer children living at home, compared to the other profiles. All profiles were significantly different regarding the composition of contract types. Profile 5 was characterized by significantly more fixed‐term contracts than other profiles. In addition, Profile 2 had the fewest fixed‐term contracts compared to other profiles. Moreover, Profile 1 had more fixed‐term contracts than Profile 3, but fewer than Profile 4, while Profile 3 had fewer fixed‐term contracts than Profile 4.

**Table 4:** Equality of means comparisons and probability estimates of demographic covariates across profiles

Profiles	Profile 1 Moderately insecure	Profile 2 Secure	Profile 3 Secure; quality‐concerned	Profile 4 Insecure; employment‐concerned	Profile 5 Insecure	Statistically significant differences between profiles
	*n* = 215 (8%)	*n* = 1777 (65%)	*n* = 406 (15%)	*n* = 177 (6%)	*n* = 154 (6%)	
Age	46.22	52.20	47.42	43.79	44.74	1: 2***, 4** 2: 3***, 4***, 5*** 3: 4***, 5**
Ph.D. tenure	11.42	15.71	12.76	9.99	9.17	1: 2***, 3*, 5*** 2: 3***, 4***, 5*** 3: 4***, 5***
Sex						
Man	0.49	0.52	0.52	0.58	0.45	4: 5*
*Woman*	0.52	0.47	0.48	0.43	0.55	
Civil status						
Single	0.16	0.14	0.14	0.09	0.19	1: 4* 2: 4* 4: 5*
Partnered	0.84	0.86	0.86	0.91	0.81	
**Children**						
None	0.43	0.53	0.41	0.36	0.45	1: 2** 2: 3***, 4***, 5*
Yes	0.57	0.47	0.59	0.64	0.55	
Contract type						
Fixed‐term	0.30	0.05	0.14	0.45	0.58	1: 2***, 3***, 4**, 5*** 2: 3***, 4***, 5*** 3: 4***, 5*** 4: 5*
Permanent	0.70	0.95	0.86	0.55	0.42	

*Notes*: Children categories: none (at home under 18 years of age); yes (children at home under 18 years of age). Civil status analysis based on *n* = 2716; Children analysis based on *n* = 2719. *p < 0.05, **p < 0.01, ***p < 0.001.

### Comparisons by health‐related indicators

**Fig. 3:**
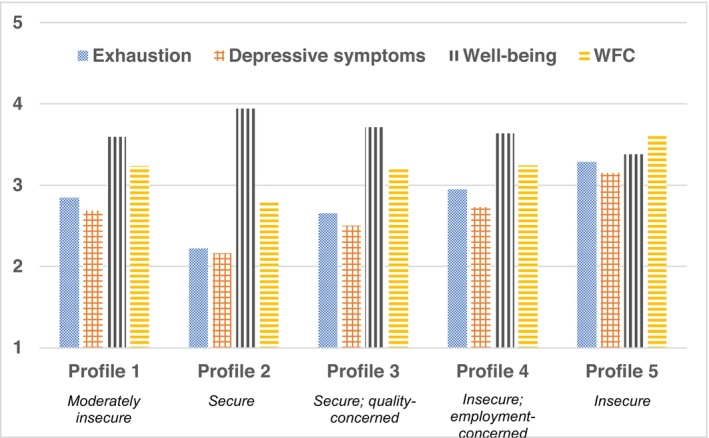
Health‐related indicators by profile membership based on scale scores.

Significant differences for exhaustion were found between all profiles with the exceptions of Profile 1 to Profile 3 and Profile 1 to Profile 4 (Table 5). Profile 2 had the lowest average exhaustion score, while Profile 5 was one point higher, with the highest average score (Hedge's *g* = 0.90; Fig. 3).

**Table 5:** Factor means and standard errors (in parentheses) and scale means and standard deviations (in brackets, in italics) for health‐related indicators by profile

Indicator	Profile 1 Moderately insecure	Profile 2 Secure	Profile 3 Secure; quality‐concerned	Profile 4 Insecure; employment‐concerned	Profile 5 Insecure	Statistically significant pair‐wise comparisons
Exhaustion	*0.40* (0.09) 2.84 [*1.23*]	*−0.22* (0.03) 2.22 [*1.17*]	*0.22* (0.06) 2.65 [*1.19*]	*0.51* (0.09) 2.94 [*1.22*]	*0.83* (0.11) 3.28 [*1.29*]	1: 2***, 5** 2: 3***, 4***, 5 *** 3: 4**, 5*** 4: 5*
Depressive symptoms	0.41 (0.06) 2.69 [0*.93*]	−.022 (0.02) 2.16 [0*.87*]	0.20 (0.05) 2.50 [0*.88*]	0.44 (0.07) 2.73 [0*.93*]	0.90 (0.08) 3.15 [0*.98*]	1: 2***, 3**, 5*** 2: 3***, 4***, 5*** 3: 4**, 5*** 4: 5***
Well‐being	−0.49 (0.06) 3.59 [*0.50*]	0.24 (0.02) 3.94 [*0.47*]	−0.22 (0.04) 3.71 [0*.49*]	−0.46 (0.07) 3.63 [*0.49*]	−0.96 (0.09) 3.38 [*0.62*]	1: 2***, 3***, 5*** 2: 3***, 4***, 5*** 3: 4**, 5*** 4: 5***
WFC	0.26 (0.07) 3.23 [*0.95*]	−0.17 (0.02) 2.81 [*1.02*]	0.22 (0.05) 3.19 [*0.93*]	0.27 (0.08) 3.24 [0*.97*]	0.65 (0.08) 3.63 [*0.98*]	1: 2***, 5*** 2: 3***, 4***, 5*** 3: 5*** 4: 5***

*Notes*: Statistically significant pair‐wise comparisons reflect differences based on factor means. *N* = 2729. WFC = work–family conflict.

* *p* < 0.05,

** *p* < 0.01,

*** *p* < 0.001.

Depressive symptoms differed significantly across all profiles except between Profiles 1 and 4. While Profile 2 was associated with symptoms indicating “being bothered a little bit in the past week,” Profile 5 was characterized by depressive symptoms occurring slightly more than “moderately” in the past week. From Profile 2 to Profile 5, there was a one‐point increase in depressive symptoms (Hedge's *g* = 1.13).

As for well‐being, there were significant differences between all profiles with the exception of Profiles 1 and 4. Profile 2 was characterized by the highest well‐being scores, while Profile 5 was associated with the lowest well‐being scores (Hedge's *g* = 1.16).

Across profiles, some significant differences emerged for WFC: Profiles 2 and 5 were significantly different from all other profiles. Profile 2 showed the lowest WFC, while Profile 5 had the highest (Hedge's *g* = 0.81). However, there were no significant differences between Profiles 1 and 3 nor between Profiles 1 and 4.

## DISCUSSION

This study sought to identify meaningful and unique profiles of JI, based on theory covering its quantitative and qualitative dimensions, and to examine these types in relation to indicators of health and well‐being within a representative sample of faculty in Sweden. Employing latent profile analysis, five JI profiles were identified: Profile 1: moderately insecure, Profile 2: secure, Profile 3: secure; quality‐concerned, Profile 4: insecure; employment‐concerned, and Profile 5: insecure. The profiles were distinguished by varying levels of quantitative and qualitative JI. While meaningful from a descriptive perspective, the overall findings also showed that increasing JI involved increasing adversity relating to both positive and negative aspects of health and well‐being, albeit with some exceptions. While this echoes existing variable‐oriented JI research, an important contribution involves using established self‐report measures to create meaningful profiles, without employing arbitrary cut‐offs to categorize high and low JI. Most respondents (65%) were secure (Profile 2), while approximately 12% belonged to the more insecure profiles (Profiles 4 and 5), suggesting that the majority of faculty in Sweden are relatively secure regarding their overall perceived JI. Still, some individuals experience more severe JI, and these individuals may be most at risk with regards to their health and well‐being.

This concurrent study of quantitative and qualitative JI contributes important knowledge regarding the extent to which one dimension of JI occurs with the other. Until now, few studies have addressed this (De Cuyper, Van Hootegem, Smet, Houben & De Witte, 2019; Ghezzi *et al*., 2022; Låstad *et al*., 2021; Urbanaviciute, Lazauskaite‐Zabielske *et al*., 2021). From theoretical and practical perspectives, it is noteworthy that the more secure profiles (Profiles 2 and 3) and Profile 1, were each characterized by fairly balanced levels of quantitative and qualitative JI (see Fig. 2b), while the insecure profiles (Profiles 4 and 5) showed greater within‐profile differences: quantitative JI noticeably exceeded qualitative JI. For those more insecure, perhaps when quantitative JI is high, this threat becomes most salient, while qualitative threats are perceived as secondary, reflecting the theorized relationship where quantitative JI may encompass qualitative (De Witte *et al*., 2015).

A recent latent profile analysis hypothesized and confirmed that high quantitative JI should coincide with high qualitative JI, whereas profiles with high qualitative JI can occur independently of quantitative JI (Urbanaviciute, Lazauskaite‐Zabielske *et al*., 2021). Our findings did not reveal any such profiles. Instead, Profiles 2 and 3 were the only ones in which qualitative exceeded quantitative, and in both cases, qualitative JI was relatively low. Such discrepancies between studies may relate to population differences: Urbanaviciute, Lazauskaite‐Zabielske *et al*., (2021) used a convenience sample of public and private sector employees, implying contextual variation, while the current sample was confined to a specific setting and population (i.e., public sector, academia). Importantly, our findings seem to suggest that among faculty, high levels of qualitative JI do not occur in isolation – they occur with high quantitative JI. Perhaps heightened perceptions of deteriorating working conditions signal that the security of the job itself is also threatened, at least among academics.

As for demographic comparisons, differences in age, Ph.D. tenure, children, and contract type by profile may be informative for understanding JI profiles in academia. Finding that the most secure profiles (Profiles 2 and 3) were also the oldest, with the longest Ph.D. tenure is reasonable given that older faculty have likely had more time to acquire merits and acclimate to academia. Moreover, the insecure profiles (Profiles 4 and 5) were also the youngest, consistent with prior research (Kinnunen *et al*., 2014) and had the shortest Ph.D. tenure. Based on the composition of contracts across some profiles, contract type seems related to JI perceptions. While objective JI was not central to this study, it is still noteworthy that the secure (Profile 2) was almost entirely comprised of permanent faculty, whereas the insecure profiles (Profiles 4 and 5) showed an equal mix of permanent and fixed‐term contracts. While this mix constitutes an important theoretical contribution reflecting the subjectivity of JI, the presence of permanent faculty in the more insecure profiles might be explained by a unique characteristic of employment in Swedish academia. Faculty, oftentimes *researchers*, can be permanently employed with the caveat that they must finance themselves with grants, sometimes referred to as a permanent employment “dependent on funding” (Swedish Association of University Teachers and Researchers & National Junior Faculty, 2021). As continued employment is contingent upon successful grant applications, this differs from other permanent positions in Swedish academia. Terminology may obscure important conditions of certain employment types, thus individuals belonging to insecure profiles with permanent contracts may in fact be faculty “dependent on funding.” This elucidates the complexities of claims made based on contract type in the Swedish academic context.

Regarding the various health‐related indicators, a noticeable pattern emerged: increasing JI was associated with greater impairment. One pattern is especially noteworthy: Profiles 1 and 4 did not differ significantly on any of the health‐related indicators. While these profiles were statistically distinguishable and meaningful from theoretical and practical perspectives, the increased level of quantitative JI was not associated with significantly different indicator scores. One explanation is that within this range of quantitative JI (approximately between 3 and 3.75, Fig. 2a), such differences are negligible. Another interpretation may relate to differences in qualitative JI being more definitive for health‐related indicators. Thus, in studies investigating both quantitative and qualitative JI, variations in health‐related indicators may be attributed to the differences in qualitative JI. Such claims are supported by latent profile analysis findings (for mental health; Urbanaviciute, Lazuaskaite *et al*., 2021) and cross‐lagged panel findings (Nawrocka *et al*., 2021).

Moreover, the results showed a consistent pattern across profiles for indicators of ill‐health (i.e., exhaustion and depressive symptoms). The difference in exhaustion scores between Profiles 2 and 5 involved a large effect (based on Hedge's *g*). While age may partially explain high exhaustion in Profile 5 (exhaustion disorder is most common in those between 35 and 44 years of age; Swedish Social Insurance Agency, 2020), our findings corroborate previous research (Kinnunen *et al*., 2014), suggesting that such high JI (e.g., Profile 5) may be taxing and contribute to exhaustion.

Depressive symptoms were lowest in Profile 2 and highest in Profile 5, also revealing a large effect. In fact, Profile 5 was associated with levels suggesting major depressive symptoms (*M* = 18.81), as the mean sum scores surpassed 17, a clinical diagnosis cut‐off (Magnusson Hanson *et al*., 2014). This suggests that individuals in Profile 5 may be particularly vulnerable for subsequent adversity.

Well‐being scores across profiles revealed the same pattern as the ill‐health indicators, yet in the opposite direction. Well‐being was lowest in Profile 5 and highest in Profile 2 and the effect of the difference in scores was large. Given that Profile 2 was also the oldest, it is possible that higher age is associated with higher well‐being. This aligns with a study of faculty across Europe showing that around age 45, employee well‐being increased, among permanent and non‐permanent faculty (Castellacci & Viñas‐Bardolet, 2021). Yet, these findings also showed that permanent faculty generally reported higher job satisfaction than non‐permanent faculty. This may explain our findings. While Profile 2 mostly included permanent faculty with higher well‐being, Profile 5 was somewhat younger, (many with fixed‐term contracts), with lower well‐being. Thus, better job security may involve better well‐being. Similarly, variable‐oriented research provides support for the relationship between JI and work‐related well‐being in academia (Urbanaviciute, Roll *et al*. 2021).

As for WFC, our findings align with prior research suggesting that JI is associated with WFC (Mauno *et al*., 2017; Voydanoff, 2005). Regarding WFC differences by profile, the same pattern as for ill‐health emerged and the effect of the difference between Profile 2 and Profile 5 scores was large. Profile 2 reported significantly lower WFC than the other profiles, which may be explained by fewer individuals in Profile 2 having children at home compared with the other profiles and by Profile 2 containing the oldest respondents, on average (Leineweber, Baltzer, Magnusson Hanson & Westerlund, 2013). However, JI may play a role in the association between profile and WFC, especially given the statistical difference in WFC scores across the other profiles, where they did not differ significantly on whether they had children at home. This suggests that high JI, when coupled with children at home, may add risking increased WFC.

Taken together, it is clear that differences across JI profiles were significantly associated with variations in health and well‐being. While prior variable‐oriented studies have shown associations between JI and adverse health (e.g., Blom *et al*., 2015), our latent profile analysis reveals that certain groups of individuals, belonging to the more insecure profiles may be more vulnerable to adversity, despite their overall resourcefulness. Our findings provide no definitive explanation of the role of demographics in these associations. Instead, the demographics serve a descriptive purpose. Importantly, groups of vulnerable individuals, also in resourceful populations, may extend beyond established categories of age and sex. Though the person‐oriented approach involves limitations, particularly regarding examining the specific impact of different variables, its benefits relate to possibilities of identifying and investigating other important groups. This, in turn, may add to the understanding of why some individuals in specific contexts are more vulnerable than others. Moreover, given that all faculty are likely to benefit from a good work environment and sustainable working conditions, identifying vulnerable groups is important for promotion, prevention and intervention, especially when resources are limited.

### Limitations

The limitations of this study include self‐reports which may entail common method bias. However, with perceived JI being a subjective perception, self‐reports seemed reasonable. Furthermore, the health‐related indicators are all considered to be adequately reported by individuals themselves, making self‐ratings most appropriate, especially given the costs associated with different types of objective measures.

Another limitation includes the link between our inclusion criteria (i.e., current employment in academia) and JI perceptions. The competition for jobs in academia is steep, and not all who wish to stay are able to. Others may wish to work outside academia. Importantly, the study design and inclusion criteria do not allow for the consideration of individuals with a doctoral degree who have had an academic position, but no longer do, regardless of their reason for no longer working in academia. Thus, our sample may be biased towards those who perceive greater security.

A related limitation regards the distribution of permanent versus fixed‐term faculty. While a representative sample of faculty was invited, the percentage of those with permanent contracts exceeded those in national statistics on employment contracts in Swedish higher education institutions. Twenty‐seven percent of all research and teaching staff are fixed‐term (Swedish Higher Education Authority, 2023), underscoring the relevance of JI research in academia, but our sample included only 13%. However, Swedish national statistics include individuals with and without doctoral degrees whereas we only invited those with a doctoral degree. This may explain such differences. Also, our inclusion criteria excluded those without current employment at a Swedish HEI as their main activity, meaning that some with part‐time employment in academia may have been excluded (e.g., those on parental leave or partially retired). While the selection of some 7500 individuals from the target population, from all public higher education institutions and subject areas resulted in an underrepresentation of fixed‐term faculty, our findings still indicate that JI perceptions are more nuanced than based strictly on high or low JI scores or contract type.

Given the representative sample of the target population (faculty with a doctoral degree, working in Swedish academia), considerations for inclusion and sample diversity were limited. Furthermore, issues of low statistical power impeded possibilities to explore differences based on ethnicity (i.e., country of origin). While Swedish law prohibits ethnic discrimination in hiring and recruitment practices, other, informal barriers may foster ethnic or cultural homogeneity. While such discussions are important, they go beyond the scope of the current work. Ultimately, the demographic makeup of our sample reflects the variation which exists among faculty with a doctoral degree, working in Swedish academia. This too brings into question the generalizability of our findings to the higher education sector, abroad. Sweden, like other Nordic countries is known for its generous welfare system, which may provide a sense of security to those faculty who experience subjective JI. In contrast, a lack of a social safety net in other countries may exacerbate any negative effects of JI. Taken together, our findings are most generalizable to the Nordic countries, where higher education systems (Frølich, Wendt, Reymert *et al*., 2018) and welfare systems (Kim, Muntaner, Shahidi, Vives, Vanroelen & Benach, 2012) are similar to Sweden.

### Strengths and future research

While variable‐oriented JI research focuses on the phenomenon, little can be derived about the individuals who experience JI from such studies. This person‐oriented study identifies groups of individuals who experience JI similarly, revealing variation within a population, and identifying those vulnerable individuals who may benefit from interventions. Future studies, implementing longitudinal designs, may help clarify whether prior JI perceptions result in job loss or occupational turnover, contributing to a furthered understanding of who stays in academia, who leaves, and why. Other avenues of research may include qualitative studies, probing the individual experiences of insecurity among faculty with different JI profiles, which may, in turn, provide a more nuanced understanding of JI experiences in relation to health and well‐being.

### Practical implications

Some vulnerable subgroups of faculty in Swedish academia may be at risk for adversity. These faculty may benefit from targeted efforts to alleviate experiences of JI. Given that perceived employability may decrease perceptions of both quantitative and qualitative JI, over time (Hu, Jiang & Chen, 2022), one viable approach to target those most vulnerable may be to boost faculty employability by means of seminars or workshops to increase awareness of comparable job opportunities, and professional networking opportunities, within and outside academia. Higher education institutions, and department leadership specifically, should strive for open and clear communication with faculty about their current work environments, and future career prospects to help mitigate faculty perceptions of JI. These efforts may serve as protective for faculty health and well‐being.

## CONCLUSION

This study aimed to explore profiles of quantitative and qualitative JI and their associations with health‐related indicators among faculty in Sweden. Five profiles of JI emerged, characterized by quantitative and qualitative JI with findings suggesting that the insecure (Profile 5) are the most vulnerable. Taken together, this study drives an ongoing discussion of the ways in which quantitative and qualitative JI coincide in various populations, and how detrimental this may be. Moreover, this study contributes to the existing person‐oriented JI research, adding that qualitative JI may be the more important dimension for health‐related outcomes. Finally, this study provides insight into the extent of perceived JI among faculty with a doctoral degree in Swedish academia. Such knowledge is important to ensure sustainable employment and working conditions.

## Author contributions

Conceptualization: AST, AR, PL; data curation: AST; formal analysis: AST, AB; funding acquisition: PL, AR; investigation: PL, AR, AST; methodology: AST, AB; project administration: AST, AR, PL; resources: PL; software: AST, AB; supervision: PL; validation: AST, AB; visualization: AST, AB; writing–original draft: AST; writing–review and editing: AST, AR, AB, PL.

## Data Availability

Data available on request due to privacy/ethical restrictions. The data which supports the findings of this study are available upon reasonable request from the fourth author. The data are not publicly available to protect the privacy and integrity of respondents.

## Endnote

^1^
A factor score is a weighted combination of responses to a set of observed items, where items which have stronger factor loadings are given more weight.
